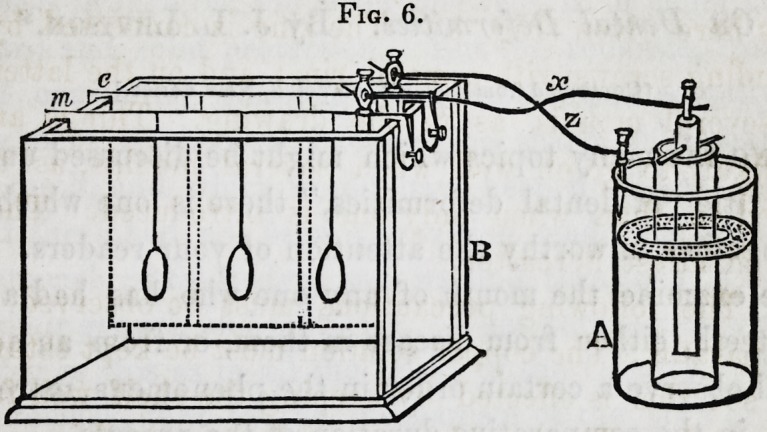# Chemistry of the Metals—Copper

**Published:** 1856-04

**Authors:** R. N. Wright


### AMERICAN JOURNAL OF DENTAL SCIENCE.

Vol. VI. NEW SERIES—APRIL, 1856. No. 2.

### ORIGINAL COMMUNICATIONS.

### ARTICLE I.

### *Chemistry of the Metals—Copper.* By Professor R. N. Wright, A. M., M. D.

(Continued from page 358, of Vol. 5, New Series.)

It was designed to have published in the October number of the Dental Journal the present article, but circumstances rendered its preparation impossible; we trust, therefore, that our seeming neglect will be duly overlooked.

We stated at the conclusion of the last number, that we would commence the present article with an account of the process for the deposition of copper by means of galvanism; but thinking a modification of that plan better adapted to the arrangement we have adopted heretofore, we will proceed first to examine the compounds of copper, natural and artificial, closing with a description of the electrotype process.

First, then, we will take the compounds with oxygen, the following account of which is to be found in "Brande's Manual."

*Copper and Oxygen—Dioxyd of Copper.*—"There is only one salifiable oxyd of copper, which, therefore, is generally regarded as the *protoxyd;* but there is a *suboxyd* or *dioxyd,* (2 cu+O,) which occurs native, and which may be formed artificially, not by the direct oxydizement of copper, but by processes, in which oxygen is abstracted from the protoxyd. The following are the methods by which this suboxyd may be obtained: 1. By heating 5 parts of peroxyd with 4 of very finely divided metallic copper; or, according to Turner, by arranging thin copper plates, one above another, with interposed strata of the black oxyd, and exposing them to a red heat carefully protected from the air. 2. By boiling a solution of acetate of copper with sugar, by which the protoxyd of the acetate is reduced to the state of suboxyd, and separates in the form of a red powder. 3. By fusing dichloride of copper with carbonate of soda, and washing and drying the residue. 4. By fusing a mixture 100 parts of crystals of sulphate of copper with 57 of crystals of carbonate of soda; when the water is expelled, the mass is reduced to powder, mixed with 25 parts of copper filings, and exposed to a white heat for about twenty minutes; the residue is then pulverized, washed and dried: it is of a red color; the tint being improved by trituration and washing. This, which is Malagutti's, is the most economical and yields the best product. (Ann. de Ch. et Ph., liv, 216.) 5. *Hydrated* suboxyd of copper is precipitated in the form of a dingy orange-colored or brown powder, when a hot solution of subchloride of copper is decomposed by potassa; if dried in vacuo, it becomes reddish brown. 6. When sulphate of copper and protosulphate of iron are dissolved in water and precipitated by an alkali, dioxyd of copper and peroxyd of iron are thrown down: the former may be separated by digestion, out of the contact of air, in ammonia, which gives a colorless solution. (Levol, Ann. de Ch. et Ph., lxv, 320.)

"When this oxyd is intensely heated out of contact of air, it frequently happens that small octëdral crystals, as well as cubes, are formed in it; the same thing was observed by Chenevix, on exposing peroxyd or hydrate of copper to a violent heat in an open crucible, without addition; a semifused mass, resembling native red copper was obtained. (Phil. Trans., 1801.) The dilute acids resolve this oxyd into metallic copper and peroxyd; it dissolves in concentrated hydrochloric acid; it also dissolves in ammonia, as will presently be explained, it is not soluble in solutions of potassa or soda.

"This compound may be regarded as consisting of 1 atom of copper and 1 of protoxyd; or, of

| | | | | Chenevix. | Berzelius. |
|---|---|---|---|---|---|
| Copper, . | 2 | 64 | 88.9 | 88.5 | 88.97 |
| Oxygen, . . | 1 | 8 | 11.1 | 11.5 | 11.03 |
| Dioxyd of copper, | 1 | 72 | 100.0 | 100.0 | 100.00 |

"Copper vessels, such as tea-urns, &c. and medals, are often superficially coated with this oxyd, or *bronzed;* it gives them an agreeable appearance, and prevents tarnish. For this purpose, two processes are resorted to: 1. The copper surface is cleaned, and then brushed over with peroxyd of iron (generally colcothar) made into a paste with water, or with a very dilute solution of acetate of copper, heat is then cautiously applied in a proper furnace or muffle, until it is found on brushing off the oxyd, that the surface beneath has acquired the proper hue. 2. Two parts of *verdigris* and 1 of sal ammoniac are dissolved in vinegar; the solution is boiled in a pipkin, skimmed and diluted with water, until it only tastes slightly of copper, and ceases to deposit a white precipitate; it is then poured into another pipkin or copper pan, and rapidly brought to boil, and the medal previously rendered bright, and *perfectly clean*, is dipped into the boiling solution, which may be most conveniently done, by placing it in a small perforated copper ladle, or wire colander, made for the purpose; the surface of the medal becomes at first black or dark-blue, and then (in about 5 minutes) acquires the desired brown tint; it must then be instantly withdrawn, (otherwise it changes color,) and washed in a stream of water, so as perfectly to remove all soluble matters from its surface; and lastly, very carefully wiped and dried. The medal is generally perfected by afterwards giving it one gentle pinch between the dies, in the coining-press. When there are many medals, each must be done separately, as they must not be allowed to touch each other, and care should be taken to rest them upon as few points of contact as possible. The bronzing liquid also, must not be suffered to concentrate by evaporation, but must be diluted if necessary, so as to keep it in a proper state, and especially to avoid all appearance of a white precipitation in it. It is better that the process should be too slowly, than too rapidly effected. Medals and voltatypes, may also be bronzed by covering their surface with plumbago, heating them to dull redness, and then brushing the surface until it acquires the desired tint."

The next oxyd we shall consider is now generally acknowledged as the *principal* salifiable base in the formation of copper salts, (formerly deutoxyd, now) protoxyd of copper ($Cu+O$.)

This oxyd is invariably formed when copper is heated in the air, and peals off as the metal cools, in the form of dark iodine colored scales. The most expeditious method of obtaining it, is to heat the copper to a cherry red, and plunge it, as soon as withdrawn from the furnace into cold water, the rapid contraction of the metal occasions the desquamation of the oxyd in considerable quantity, and when cold, we have but to reduce it to fine powder, expose it to a red heat, agitating it at the same time, so that there shall be free atmospheric contact, and we obtain the desired result.

With reference to this oxyd, Brande has the following, p. 814.

"*Protoxyd of copper* is black, or bluish or brownish-black: its specific gravity is 6.4. Before the blow-pipe, it fuses when intensely heated, by the tip of the flame, upon charcoal: by the interior of the flame, it readily affords a globule of metal. It is very readily decomposed at a dull red heat, by hydrogen and by carbon, and consequently also by organic substances; hence its use in organic analysis; it is very hygrometric, and for this reason, if weighed while hot, it generally augments in weight after cooling, in consequence of the absorption of aërial moisture. It is insoluble in water, but it dissolves in, or combines with, the greater number of the acids, and is the basis of all the common salts of copper. When alkalies are dropped into its solutions, they throw it down, as a bulky blue hydrate, which, however, is not permanent at a boiling heat, but becomes black and anhydrous.

"This oxyd of copper is not soluble in the liquid fixed alkalies, but when carbonate of potassa, or of soda are fused with it, it expels carbonic acid, and combines to form a blue or green compound. It communicates a green, and sometimes a blue tint to vitreous compounds; and Sir H. Davy has shown that it is the basis of certain colors used by the ancients, which had been supposed to contain cobalt. (Phil. Trans., 1815.) It consists of,

| | | | | Proust. | Berzelius. | Gay Lussac. |
|---|---|---|---|---|---|---|
| Copper, | 1 | 32 | 80 | 80 | 80.13 | 80.28 |
| Oxygen, | 1 | 8 | 20 | 20 | 19.87 | 19.72 |
| | 1 | 40 | 100 | 100 | 100.00 | 100.00 |

This oxyd is sometimes considered as a *deutoxyde*, ($Cu+O_2$,) and the *dioxyd*, as a *protoxyd;* in this case, 64 becomes the equivalent of copper. Gmelin and Berzelius, however, regard the salifiable oxyd as the protoxyd, or as constituted of 1 atom of copper, and 1 atom of oxygen. Turner also has adopted this view, and urges in favor of it, 1st, That the general characters of a suboxyd belong to the red oxyd. 2d, That the equivalent of copper, deduced from its specific heat (p. 163) is, 32; and 3d, that the salts of black oxyd of copper are isomorphous, with those of the protoxyd of iron; which gives a strong presumption, that those oxyds possess the same atomic constitution. To these arguments it may be added, that the electro-chemical equivalent of copper appears to be 32."

The following alkaline precipitate, commonly used as a coloring ingredient in paper staining, &c., is thus spoken of by the author above quoted:

"*Hydrated Protoxyd of Copper*, as thrown down from a solution of sulphate of copper by potassa or soda, is at first of an agreeable blue color, but this soon changes to green, especially if it be dried; when it is used as a pigment or color for paper staining, it is rendered more permanent by mixing it with glue or size, and chalk or alumina are sometimes added: it, however, soon acquires a green tinge. Dumas gives the following process for preparing a blue color with this hydrate: 6 parts of sulphate of copper, and 3 parts of chloride of calcium, are dissolved in separate portions of water; the solutions are then mixed, and when the sulphate of lime has subsided, the solution of chloride of copper is poured off and mixed with cream of lime, containing 1½ parts of quick lime; the mixture is well agitated, and the greenish precipitate which falls, and which is an oxychloride of copper, being well drained, is ground up with a fourth part of lime, and a fourth part of potash of commerce, so as to form a mixture of proper consistency. When this paste is put into bottles, a fourth part of sal ammoniac, and half a part of sulphate of copper, are added to improve the color, which, however, cannot be depended on, as it is apt to become green when dry, so that it is generally sold in the form of paste, to avoid the risk of desiccation."

There is another oxyd of copper, abounding in the vicinity of Cornwall, Great Britain, having a specific gravity between 5 and 6, with a reddish or drab color, commonly called "tile ore," which occurs massive, or in six, eight or twelve sided crystals, but not sufficiently important to demand especial notice.

*Sulphuret of Copper.*—The first of these compounds to which we would direct attention, is that which is most abundant and most used in the manufacture of copper for commercial purposes; viz. the ferruginous sulphuret, or copper pyrites, as it is commonly called.

We are probably speaking within bounds, in saying, that more than half the copper of commerce is procured from this combination; some specimens are remarkably beautiful, being highly iridescent; indeed, so singularly beautiful is the play of colors in some specimens that the term "pavonine"* sulphuret has been used. The usual form in which it appears is that of tetraëdral crystals, and in most specimens, we find nothing but iron, copper and sulphur, but occasionally, there is a small quantity of lead, and a minute portion of arsenic.

*Sulphuret of Copper.*—Cu+s. When sulphuretted hydrogen

* From a fancied resemblance to the gorgeous colors of the peacock.

gas is passed through a solution of some salt of protoxyd of copper, a dense precipitate is deposited, which, upon examination, turns out to be a sulphuret of copper, having colors varying with its condition of dryness, being brown when first deposited, gradually changing to black, and greenish-black as it dries. Its chemical constitution is as follows:

| | | |
|---|---|---|
| Copper, . . . | 1 atom | 32 |
| Sulphur, . . . | 1 " | 16 |
| Protosulphuret of copper, | 1 " | 48 |

*Disulphuret of Copper.*—2 Cu+s. This compound is produced whenever a mixture of copper turnings and sulphur are heated together, until the sulphur melts; as soon as this happens the action between the substances becomes quite violent; the copper assuming a bright red heat, and combination occurring with the formation of a black brittle mass, found upon examination to be a disulphuret of copper, and to be constituted as follows:

| | | |
|---|---|---|
| Copper, . . . | 2 | 64 |
| Sulphur, . . . | 1 | 16 |
| Disulphuret of copper, | 1 | 80 |

*Combinations of Copper and Chlorine.*—There are two of these occurring when chlorine and copper come into contact; viz. the *dicloride* and the *potochloride*, which are thus described by Brande.

*"*Dichloride of Copper*—(2 Cu+Cl)—was first described by Boyle in 1666, under the name of *rosin of copper.* It was afterwards examined by Proust, who called it white muriate of copper. (Ann. de Chimie, xxviii, 218.) It may be obtained by exposing *copper filings* to the action of chlorine not in excess; or by evaporating the solution of dioxyd in *hydrochloric acid*, and heating the residue in a vessel, with a very small orifice; or by heating the *protochloride* in the same way. It is also the residue of the distillation of a mixture of 2 parts of *corrosive sublimate*, and 1 of copper filings. When *protochloride of tin* is added to any soluble salts of copper, or when *copper filings*

*Brande, p. 815.

and the *protoxyd* are digested in hydrochloric acid, this *dichloride* is also formed. It is insoluble in water, but soluble in hydrochloric acid, from which potassa throws down the *hydrated dioxyd.* When water is added to its *hydrochloric solution,* it is thrown down in the form of a white granular hydrate: its color varies, being generally dark brown; but if fused and slowly cooled, it is yellow, translucent and crystalline: it must be preserved out of contact of air. It consists of

| | | | J. Davy. |
|---|---|---|---|
| Copper, . . | 2 | 64 | 64 |
| Chlorine, . . | 1 | 36 | 36 |
| Dichloride of copper, | 1 | 100 | 100 |

With reference to the compound of copper and chlorine commonly known as Brunswick green, the same author has the following:

"*Hydrated Dichloride of Copper—Submuriate of Copper.*—When moistened dichloride of copper is exposed to air, it acquires a greenish-white color, and becomes converted into a compound of chloride and oxyd of copper, which has been termed, *submuriate of copper,* or *Brunswick green;* the same compound may be formed by adding *hydrated oxyd of copper* to a solution of the chloride, or by exposing to the atmosphere slips of copper, partially immersed in *hydrochloric acid.* This compound consists, according to John Davy, of 4 atoms of *protoxyd of copper,* 1 of *hydrochloric acid* and 3 of water. Protochloride (Cu+Cl) is obtained, under ordinary circumstances, by throwing copper into *hydrochloric acid*; the copper is dissolved, and the residue must be obtained by evaporation to dryness at a moderate heat. Another method has been proposed by Dumas, which in substance is as follows: he proposes to decompose *sulphate of copper* by mixing with it *chloride of calcium,* he then removes sulphate of lime by filtration, and subsequently evaporates the fluid to the consistence of syrup and obtains the chloride by solution in alcohol.

When this compound is perfectly formed and is free from water, it has a yellowish-brown appearance, but becomes blue by exposure to the atmosphere. When a solution of potassa is added in moderate quantity, a green compound is deposited, which is most probably a *subchloride;* heated to redness, it becomes converted into *dichloride*, by losing a portion of its chlorine; and whenever allowed to come in contact with *ammonia*, in the form of gas, will take up, it is said, 56 per cent., and become changed into a bluish colored substance and quite pulverulent; this chloride has the following chemical constitution:

| | | |
|---|---|---|
| Copper, . . . | 1 | 32 |
| Chlorine, . . . | 1 | 36 |
| Protochloride of copper, | 1 | 68 |

There is another compound belonging to this class, and found native in some districts, the *atacamite* of mineralogists; we will not attempt to describe it, but merely give below the result of Dumas' analysis, according to which it consists of

| | | | |
|---|---|---|---|
| Protoxyd of copper, | 3 | 120 | 53.5 |
| Protochloride " | 1 | 68 | 39.5 |
| Water, . . | 4 | 36 | 16.0 |
| | 1 | 224 | 100.0 |

*Cyanurets of Copper.*—"*Hydrocyanic acid and hydrocyanate of potassa, throw down a white curdy precipitate in the solution of *dichloride of copper*. A similar compound is obtained by the action of *dilute hydrocyanic acid*, upon *hydrated dioxyd of copper*, it is soluble in ammonia and the solution is colorless; it is also soluble in *hydrochloric acid*, and precipitated by dilution and by potassa; it combines with other metallic cyanurets, forming a class of *cupro-cyanurets*. When *hydrated oxyd* or *carbonate of copper* is digested in dilute hydrocyanic acid, a yellow powder is formed, insoluble in water, soluble in *hydrochloric acid* and again precipitable by dilution."

*Ferrocyanuret of Copper*—may be formed by adding ferrocyanuret of potassium in solution, to a solution of one of the soluble salts of copper; it falls to the bottom of the glass, in the shape of reddish or chocolate colored gelatinous precipitate.

* Brande, (Eng. Ed.,) p. 825.

The reaction in this instance is exceedingly prompt, and a very small quantity of copper will be revealed, thus making ferrocyanuret of potassium useful as a test for the presence of copper in certain conditions.

*Fluoride of Copper.*—The mixture of *hydrofluoric acid* and *hydrated dioxyd of copper*, gives a fusible compound, having a dark color while hot, but changing to red as refrigeration progresses; the process of evaporation being conducted so that the air cannot obtain access to it.

*The Sub and Protoiodides of Copper* have not been sufficiently examined to render an account of them in this place, either interesting or useful.

*Sulphate of Copper.*—There are many processes by which this compound may be formed, but the mention of one or two will be sufficient for our purpose; when copper, in contact with atmospheric air, is exposed to the action of sulphuric acid, to which a small quantity of water has been added, the salt is formed in abundance, it is also formed when a similar mixture is exposed to the action of heat, but perhaps the most common method of obtaining it is to expose the roasted sulphuret to the air and moisture, here it is formed abundantly, though for any except the most ordinary purposes, as prepared in this way, it is wholly unfit, containing a considerable per centage of impurities.

When pure, sulphate of copper presents a most beautiful example of crystallization; its crystals are rhomboidal and have a most beautiful blue color, they almost entirely resist the action of the atmosphere, being (when well formed) but slightly efflorescent. Under the action of heat at about 400° Fahrenheit, it crumbles down into a white powder, but if this be allowed to reabsorb moisture by exposure to the air, it will speedily regain its blue color; a continued red heat will wholly decompose it, evolving *anhydrous* sulphuric acid, while the oxyd of copper remains.

*"Sulphate of copper is much used in the arts, as a source of several blue and green colors. It is also employed by dyers

* **Brande, (Eng. Ed.) p. 822.**

and calico printers, and is an ingredient in some kinds of writing ink. It has also been used to prevent smut in corn, by steeping the grain in a dilute solution of the salt; (Quart. Jour. xvi, 156,) and minute quantities of it are occasionally added to bread, (in Paris especially,) to improve its color and quality. (Archives Gén. de Médecine, xxi, 145.) In medicine it is resorted to as a powerful emetic; and in very minute doses, as a tonic. It is also a valuable external application as an astringent, or when undiluted, as a styptic and caustic. It may be employed to prevent dry rot, by steeping timber or planks in its solution, and it is a powerful preservative of animal substances, which, when imbued with it and dried, remain unaltered. The waters of copper mines, often hold it in solution, and, when decomposed by immersing in them pieces of *iron*, yield precipitated metallic copper, (copper of cementation.) The precipitation of metallic copper during the electro-chemical-decomposition, of an aqueous solution of this salt, is now applied very extensively and successfully to the production of duplicates of medals, bank note plates," &c.

Anhydrous sulphate contains:

| | | | | Berzelius. |
|---|---|---|---|---|
| Oxyd of copper, . . | 1 | 40 | 50 | 50.9 |
| Sulphuric acid. . . | 1 | 40 | 50 | 49.1 |
| Anhydrous sulphate of copper, | 1 | 80 | 100 | 100.0 |

The common rhomboidal crystals contain:

| | | | | Proust. | Berzelius. |
|---|---|---|---|---|---|
| Oxyd of copper, . | 1 | 40 | 32 | 32 | 32.13 |
| Sulphuric acid, . | 1 | 40 | 32 | 32 | 31.57 |
| Water, . . . . | 5 | 45 | 36 | 35 | 36.30 |
| Crystals of sulphate of copper, | 1 | 125 | 100 | 100 | 100.00 |

*Nitrate of Copper.*—Copper is very oxydizable in nitric acid, accordingly if the diluted acid be poured upon fragments of copper, brisk action immediately commences, the oxyd of copper is formed at the expense of a portion of the acid, evolving the deutoxyd of nitrogen, the undecomposed acid uniting with the oxyd and forming a deep blue solution, which contains the *ni-*

*trate of copper;* this may now be obtained in prismatic crystals, either by spontaneous or artificial evaporation. It is hard to keep in a crystalline form, from the fact that it contains a large amount of water, it is very soluble in water, and will also dissolve freely in alcohol, imparting to the flame of burning alcohol, a rich green tint, a similar tint is also imparted to the blowpipe flame. Sprinkled with water and wrapped in tin-foil, violent action immediately commences, attended with considerable heat and sometimes also with light, the nitric acid at the same time being decomposed, with the copious evolution of hyponitric acid.

A salt which detonates slightly on the application of heat, is formed when ammonia is added in excess to the solution of nitrate of copper; the first addition simply throws down the hydrated oxyd, an additional quantity will dissolve this, giving us the crystallizable solution in question, *ammonio-nitrate of copper.*

Nitrate of copper is constituted as follows:

| | | | |
|---|---|---|---|
| Oxyd of copper, | 1 | 40 | 42.6 |
| Nitric acid, . | 1 | 54 | 57.4 |
| | – | — | —— |
| Nitrate of copper, | 1 | 94 | 100.0 |

*Subacetate of Copper—Common Verdigris.*—This substance is abundantly formed by exposing plates of copper to the action of acetic acid, they quickly become corroded and the deposit is from time to time removed; a more common method practiced now is to alternate pieces of woolen cloth and plates of copper, the former saturated with acetic acid; as the plates become covered with acetate, the deposit is removed.

This salt consists of,

| | | | |
|---|---|---|---|
| Oxyd of copper, . | 2 | 80 | 43.24 |
| Acetic acid, . | 1 | 51 | 27.57 |
| Water, . . | 6 | 54 | 29.19 |
| | – | —— | ——— |
| Sub-acetate of copper, | 1 | 185 | 100.00 |

*Acetate of Copper, Crystallized Verdigris.*—This salt seems to have been known from a very remote period, and is formed by dissolving the verdigris of commerce in acetic acid and crystallizing; this process is carried on slowly, and most commonly by suspending in the solution small pieces of sticks, straws, &c. The crystals are oblique and rhombic, having a blue-green color, which will scarcely dissolve in alcohol, but readily in 5 or 6 parts of water; if ignited with combustibles a green tinge is imparted to the flame.

This salt consists of:

| | | | |
|---|---|---|---|
| Oxyd of copper, | 1 | 40 | 39.2 |
| Acetic acid, | 1 | 51 | 49.9 |
| Water, . | 1 | 9 | 10.9 |
| Acetate of copper, | 1 | 100 | 100.0 |

*Carbonate of Copper.*—When the solutions of carbonated fixed alkalies are added to hot copper solutions, a massive hydrate is the result, which has a green color and is sold in the shops under the title of *green verditer* and used as a pigment. Copper sulphate in solution precipitated by carbonate of potash, yields a similar result of a blue color, also used as a pigment, and called blue verditer. The green carbonate of copper occurs abundantly native; and is known to mineralogists as *malachite,* it is rarely found crystallized, but is generally a very beautiful mineral.

*Chloride of Copper*—containing one equivalent of each of its constituents, is formed by passing chlorine gas, through a mixture of water and oxyd of copper, it is not important.

*Citrate of Copper.*—*Citrate* acid added to a solution of either sulphate or nitrate of copper, yields a pale blue citrate of copper, which is also unimportant.

*Deposition of Copper by Galvanism—Preparation of Moulds.*

*"20. I can very reasonably conclude that the amateur will commence his experiments on the smaller works of art; and, as a knowledge of the mode of manipulation to copy these will, with a little practical experience, easily pave the way towards accomplishing greater things, I shall dwell principally on the art of

* **Walker's Electrotype Manipulator.**

copying medals, medallions, seals, &c., taking the reader with me through the entire process.

"21. There are many materials fitted for forming moulds; of these—*fusible metal*, *wax*, *stearine*, *gutta percha*, and a composition whose base is *spermaceti*, are mostly used. The first is applicable to all medals of ordinary size—the others to plaster medallions and larger medals.

"22. *Fusible Metal.*—This is an alloy, consisting of bismuth, tin, and lead; it melts at a low temperature—a few degrees below that of boiling water; and has been used as a philosophical toy, in the form of spoons, which melt in hot tea. For the latter purpose, it generally contains a small portion of mercury. Since the discovery of electrotype, it has been prepared for that process without mercury.

"23. The proportion of the different ingredients in a pound of this alloy is:

| | oz. |
|---|---|
| Bismuth, . . . | 8 |
| Tin, . . . . | 3 |
| Lead, . . . . | 5 |
| | 16 = 1 ℔. |

These should be melted together in a *clean* iron ladle, taking care to keep it on the fire no longer than is necessary to produce the complete liquefaction of the several ingredients. When melted, pour the metal on a stone or marble slab in drops. Then, after having *rubbed the ladle clean* with coarse paper, return the pieces of metal, re-melt them, and pour them out in drops as before. A third melting will insure the ingredients being well mixed. To retain the metal in a fit condition for use, the ladle must be frequently rubbed clean; and must always be removed from the fire *as soon* as its contents are melted. The former insures a bright surface to the mould; the latter preserves the alloy from waste by oxydation.

"24. *To make a Mould in Fusible Metal.*—Melt some in the iron ladle, and pour it on a slab; then, from the height of two or three inches, drop on it the medal to be copied, taking care that the medal is COLD. In a few *seconds* the metal will be solid, and may be placed to cool; when it is cold, either with or without a few slight taps, the two will separate: and, if proper care has been taken, an *exceedingly sharp* mould will be obtained. The novice must not, however, be disheartened if his first attempts to obtain good moulds fail: for there are so many little accidents which may happen, that the most practiced manipulator may have to repeat his attempts. A slight shake of the hand may drop the medal irregularly; too much sunk, for instance, on one side. A film of oxyd may rest on a portion of the surface of the melted metal, and render the corresponding portion of the cast *dull.* Dull looking moulds must always be rejected; for so minutely correct is the process of electrotype, that the dullness of the mould will be transferred to *every* copy made from it. Even if an original medal be incautiously handled, the slight trace of a finger-mark will be transferred to the mould; and thence to the electrotype copies.

"25. The fusible metal will not always pour into a *round* mass, to receive the medal: unless the slab is perfectly level, it runs into a stream. This is a great inconvenience, but may be remedied by having a shallow cavity (saucer fashion) made in the marble; or by using any article of *earthenware*, which the kitchen or the laboratory may furnish, suited to the purpose. I have been in the habit of using the brown stoneware saucers, in which blacking is sold; and in them have produced some of the best moulds. They are to be inverted, and the metal is to be poured on them.

"26. *Clichée Moulds.*—The following is the mode adopted on the continent for obtaining the beautiful cast of the French medals, which are so much admired. These casts are in a fusible alloy, containing *antimony*, as well as the other ingredients (§ 23.) The composition is:*

| | |
|---|---|
| Bismuth, . . . . . . . | 8 parts. |
| Tin, . . , . . . . | 4 |
| Lead, . . . . . . . | 5 |
| Antimony, . . . . . . . | 1 |

* Vide Proceed. Elec. Soc. part ii, p. 90, Aug. 17, 1841.

The metal should be repeatedly melted and poured into drops, until they are well mixed.

"27. A block of wood is then turned into a shape similar to that of a button-die, into one end of which is worked a cavity, the size of the medal to be copied, and *not quite so deep* as its thickness; in this cavity the medal is placed; should it not fit tightly, a circle of paper is pressed in with it; the medal, being thus firmly mounted, is to be copied in the following manner:

"28. A sheet of smooth cartridge-paper is fixed, by drawing-pins or otherwise, within side a box having sides about four inches high, which slope inwards in order to prevent the metal from being scattered away; the part to be used is very slightly oiled with a single drop of oil; on this is poured some of the prepared alloy, which should be removed from the fire as soon as melted (§ 23.) The metal is then stirred together with cards until it assumes a pasty appearance, and is on the eve of crystallizing; if, at this stage, the surface should appear defaced with dross, one of the cards must be passed over it lightly and speedily; should no dross appear, this part of the process may be omitted. The die containing the medal must then be held firmly in the right hand, and be struck gently and steadily upon the solidifying metal. Should an assistant be in hand to aid in this, it will be as well; for sometimes during the brief interim, while the card is being exchanged for the die, the exact moment is lost, and the mould is imperfect. When one stirs the metal, and the other is prepared with the die, the operation can be timed to a nicety. When an assistant is not at hand, the die should be placed within reach of the right hand, with the medal downwards. A little ingenuity will readily suggest the construction of a press, by which this part of the process could be accomplished. Large medals are moulded without the die, by dropping them in a sidelong direction upon the solidifying metal.

"29. The beauty and perfection of moulds thus obtained will amply repay the trouble of producing them—though I am not quite justified in using the word "trouble"—for by this mode, with ordinary care, two out of every three casts are perfect; besides, therefore, the economy of time, the saving in the reduced oxydization of metal is thus of no inconsiderable importance.

"30. This method of producing moulds is not confined to obtaining them from medals, which melt at a *high* temperature; they may be obtained from the common *soft*, *white* metal, with little danger of damaging the original. They may also be obtained from the metallic casts, which are extant, of the French medals of Andrieu, &c. Moreover, if the fusible mould itself be cut round and fitted into the block in place of the medal, it may be employed as a die; and casts, *perfect* casts, equal in respect to fidelity, and similar to the original medal, may be obtained.

"31. Having obtained a mould, varnish the back and edge—and also a portion of the front, when the surface of the mould around the impression is larger than necessary. The best varnish is good sealingwax, dissolved in spirit of wine; but for immersion in the cyanide solutions to be described hereafter (§ 95,) wax, or, which is better, pitch must be used. It will now be ready for use, and it is to be attached to a copper wire. The end of this wire must be *quite clean;* the wire is placed across the flame of the candle, with the clean end beyond the flame; it is to be touched with a piece of rosin, and pressed on the edge of the mould. The mould will instantly melt to receive it, and in a few seconds it will be cold and firmly fixed. The moulds should be wrapped in paper, if they are not intended for immediate use.

Fig. 3.

Fig. 4.

"51. *Single-cell Apparatus.*—The annexed wood-cut (Fig. 3) represents the single-cell apparatus in its complete form. *z* is a rod of amalgamated zinc, *m* the mould, *w* the wire joining them, *c* the copper solution, *p* a tube of porous earthenware, containing a solution of acid and water. To put this in action, pour in the copper solution, fill the tube with the acid water, and place it as shown in the figure. *Last* (§ 75) of all, put in the bent wire, having the zinc at one end and the mould at the other. Another form of this apparatus is here given (Fig. 4.) The zinc is connected by a wire and binding screws with a metal rim; and on the latter can be hung several moulds, as in the drawing. Things are much more likely to go on well when several moulds, as thus, are operated on, than when only one is introduced. The reason will be manifest hereafter.

"52. The following precautions must be observed in using this apparatus: The copper solution must be kept saturated, or nearly so; this is effected by keeping the shelf well furnished with crystals. The mould must not be too small in proportion to the size of the zinc. The concentrated part of the solution must not be allowed to remain at the bottom. In the latter case, the copy will be irregular in thickness—in the former, the metal may be a compact brittle mass; or may be deposited in a dull red, a violet, or a black powder. The nature of these several depositions will be elsewhere alluded to (§ 62, &c.;) so will also the relative proportions of the zinc, &c. (§ 78.)

"56. *Battery Apparatus.*—A valuable improvement was devised in Russia, by Professor Jacobi,* and in England by a member of the Electrical Society, Mr. Mason.† It consists in using a decomposition cell, analogous to that already described (§ 11.) The constant voltaic pair (§ 17,) of copper and zinc is used as the generating cell. To the end of the wire attached to the copper is fastened a *plate of copper:* to the end of the wire attached to the zinc is affixed *the mould.* The sheet of copper and the mould are placed face to face in the decomposition cell. This arrangement will be better understood from the annexed figure. A, is a cell of Daniell's battery (§§ 17, 50;) B, the decomposition cell, filled with the dilute acid solution of sulphate of copper; *c*, the sheet of copper to furnish a supply; *m*, the moulds to receive the deposit. To charge this, pour in

* Vide Jacobi's Galvano-Plastic.

† Vide Proceedings of the Electrical Society, April, 1840, p. 203.

the several solutions: hang a piece of copper on the brass rod *c;* connect this rod with the copper of the generating cell by the wire *z;* and the other rod *m*, with the zinc, by the wire *x;* *then,* and not *till* then (§ 51,) hang the moulds on the rod *m.*

Fig. 6.

"57. *Solutions.*—The solution used in this decomposition cell or depositing trough, greatly depends on the battery or power employed; with a cell of Daniell's constant battery, a solution of about 2 sulphate of copper by measure, and 1 acid water (1 acid + 9 water,) is undoubtedly the best. When less power is employed, a little acid in addition is found to be advantageous.

"Professor Von Kobell, instead of mixing acid water with the saturated solution of sulphate of copper, adds solutions of Glauber's salt, or of potash alum, or of nitrate of potash; by which means he obtains deposits of very malleable copper. Glauber's salt appears to be the best; it renders the solution more conductible, and is not itself decomposed by such feeble currents, as are here in use; while its solution will take up as much sulphate of copper as common water does. Two of saturated solution of sulphate of copper, and one of sulphate of copper in solution of Glauber's salt, are stated to be good proportions.

"To the ordinary solution of sulphate of copper, the Messrs. Elkintons add caustic potash or soda in small quantities, until the precipitate is no longer redissolved by the solution, and they thus obtain a solution for the precipitating trough which gives up a greater quantity of copper for a given battery action, and gives it up also in less space of time."

## Figures and Tables

**Fig. 3. f1:**
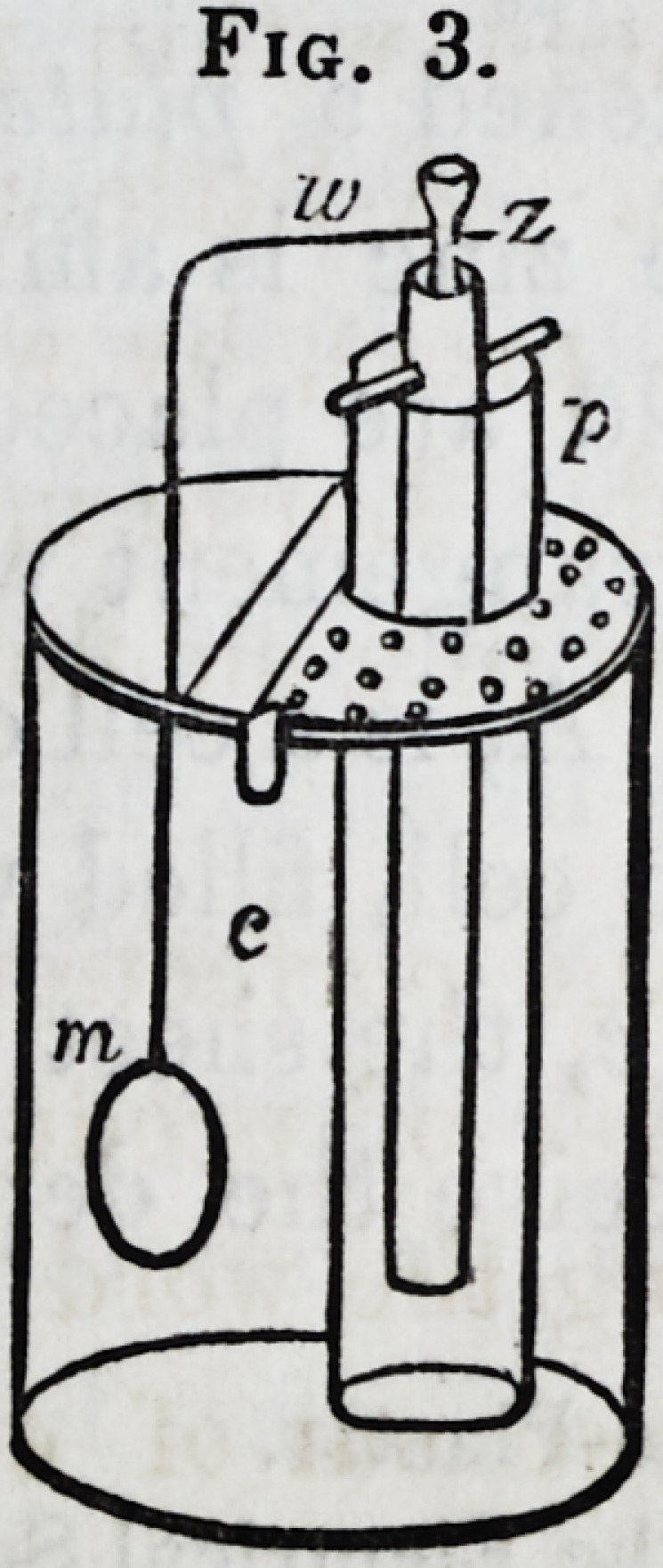


**Fig. 4. f2:**
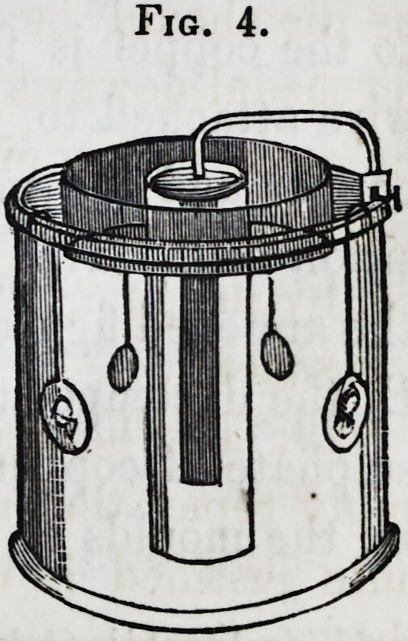


**Fig. 6. f3:**